# On the Formation of AlNiCo Nano-Quasicrystalline Phase during Mechanical Alloying through Electroless Ni-P Plating of Starting Particles

**DOI:** 10.3390/ma12142294

**Published:** 2019-07-18

**Authors:** Seyedmehdi Hosseini, Pavel Novák

**Affiliations:** Department of Metals and Corrosion Engineering, University of Chemistry and Technology Prague, 166 28 Prague, Czech Republic

**Keywords:** nano-quasicrystalline, phase transformation, electroless Ni-P plating, particles

## Abstract

A new strategy was applied to develop nano-quasicrystalline phase in well-known AlNiCo ternary system. This approach was based on electroless Ni-P plating of the starting powders and subsequent ball milling in a protective atmosphere without additional annealing or sintering processes. Microstructural evolution and phase transformation of both raw and coated particles were analyzed by scanning electron microscope (SEM) and X-ray diffraction (XRD), respectively. After 360 min of mechanical alloying, the peaks demonstrating the formation of nano-quasicrystalline phase appeared in XRD pattern of the coated powders, while those in mechanically alloyed raw powders remained mostly unchanged. The formation of nano-quasicrystalline phase in the vicinity of the primary elements was also confirmed by the corresponding selected area diffraction patterns, and images generated by transmission electron microscope (TEM).

## 1. Introduction

There are various techniques for the preparation of quasicrystalline (QC) alloys; namely liquid phase quenching, sputter or vapor deposition, ion beam techniques, and solid-state reaction during interdiffusion (alloying). Among the alloying processes developed for producing the stable and metastable powders, mechanical alloying (MA) is significant due to its simplicity and wide applications [1].

To date, there have been numerous publications dealing with the preparation of QC phases using mechanical activation followed by high-temperature long-term annealing or sintering methods in different binary and ternary systems [2,3]. Nevertheless, the direct formation of a QC phase using MA was originally reported by Eckert et al. [4] in the Al-Cu-Mn system. They illustrated that a powder mixture with the composition of Al_65_Cu_20_Mn_15_ transforms to a QC phase by a planetary mill after 90 h. Following this report, several investigators published few reports on the formation of QC phases upon long-term MA in elemental powder systems, such as Al-Mn-Si-Ge (97 h), Mg-Al-Zn (60 h), Al-Pd-Mn (30 h), and Al-Cu-Fe (20 h) [5].

Formation of a stable decagonal QC phase in conventionally solidified Al-Ni-Co system was initially demonstrated by Tsai et al. [6]. Korchagin et al. [7] applied mechanical activation for 30 s to enable self-propagating high-temperature synthesis of the quasicrystalline in this system. Yadav et al. [3] used the pre-alloyed quasicrystalline Al-Ni-Co prepared by conventional melting and solidification for studying the phase stability and structural modification induced by mechanical milling and subsequent annealing. To the best of our knowledge, however, no data on the direct formation of QC phases upon ball milling of the Al-Ni-Co system are available.

This paper explains how the electroless Ni-P plating (ENP) assists the Al-Ni-Co powder system to be directly transformed to QC phases in a relatively short period of time during MA.

## 2. Materials and Methods

The raw powder mixtures for MA contained Al (particle size < 20 µm, 99.9% purity, Strem Chemicals), Co (particle size < 5 µm, 99.9% purity, Sigma-Aldrich, St. Louis, MO, USA), and Ni (particle size < 5 µm, 99.9% purity, Sigma-Aldrich). The Ni particles were almost spherical, while the Al and Co had irregular shapes.

The electroless Ni-P plating of Al and Co powders was performed based on the instruction described for bath No. 4 of Table 1 in Reference [8]. The pretreatment operation of the powders before immersing into the electroless plating solution was comprised of two steps; namely, surface cleaning by an acetone solution, and surface activation by a diluted mixture of nitric and hydrofluoric acids for 15 min. The amount of Ni coated on the powders was 14 wt% of the mixture in total. Therefore, the rest was compensated by the addition of the raw Ni powders to the mixtures.

The mixture with the Al_70_Ni_15_Co_15_ chemical composition was subjected to MA by a planetary ball mill in various times. It was performed by steel balls 8 mm in diameter in a stainless steel vial at the speed of 400 rpm under argon atmosphere using Retsch PM-100 (Haan, Germany).

Microstructural analysis was carried out by SEM (Tescan VEGA 3-LMU, 20 kV, Brno, Czech Republic) equipped with an EDS and TEM (JEOL JEM 2200 FS, 200 kV, Tokyo, Japan). The phase formation was studied by X-ray diffraction using a PANalytical X’Pert Pro device (Panalytical, Almelo, The Netherlands) with Cu–Kα radiation.

Vickers microhardness (HV 0.01) of the specimens was measured using Hannemann hardness tester installed on Carl Zeiss Neophot 2 metallographic optical microscope (Carl Zeiss, Jena, Germany). Ball milled powders were compacted by cold pressing under 70 kN force for the microhardness measurement.

## 3. Results

**Figure 1:**
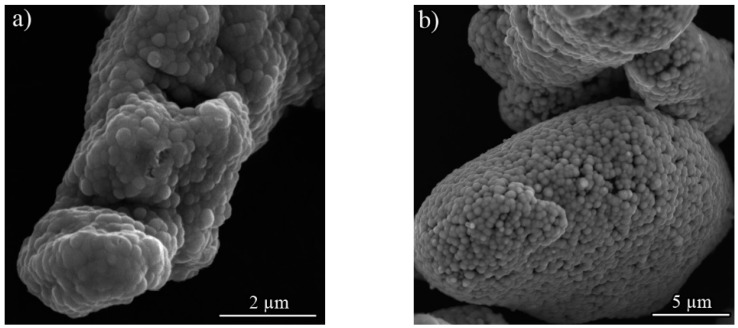

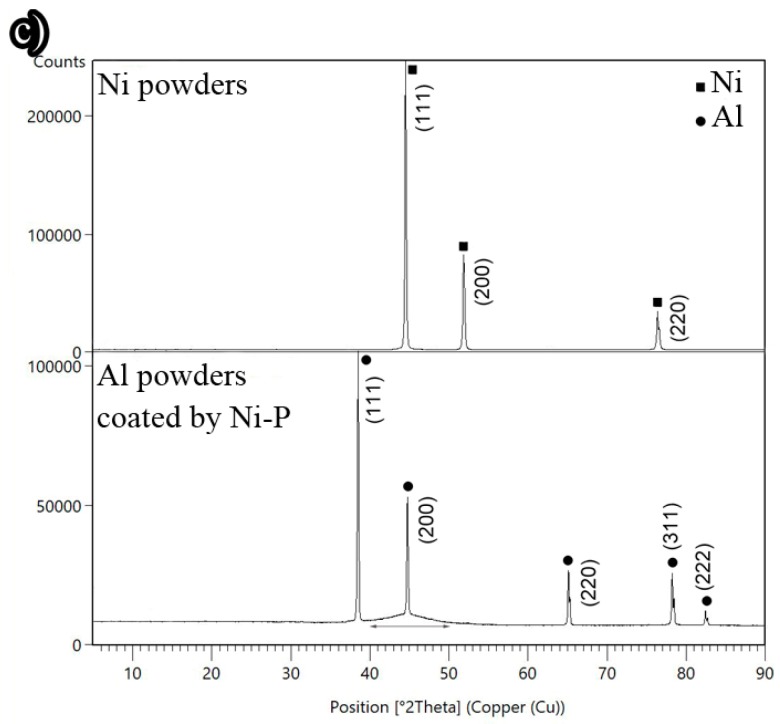
SEM micrographs of the electroless Ni-P coating for (**a**) Co and (**b**) Al particles; (**c**) XRD patterns of the ENP coated Al and metallic Ni powders.

Figure 1a,b represents the surface morphologies of the ENP coated Co and Al powers, respectively. The surface of the powders was uniformly coated by the Ni-P nodules, while the typical cauliflower structure was rarely observed on the deposition. The formation of such a pea-like structure was attributed to the deposition parameters of the electroless bath, which were adjusted so that the minimum thickness of the coating is formed on the substrate. It minimizes the required time of milling for the next step. Figure 1c shows XRD patterns of the ENP coated Al particles and metallic Ni. It was observed that the sharp peaks corresponding to Ni element vanish in the XRD pattern of the Ni-P coated Al. However, the deposited Ni-P changes the shape of (200) reflection in Al coated particles, indicating the formation of a semi-amorphous profile with a wide angular range of approximately 40–50° (2θ) along with (111) reflection from nickel (marked by arrow).

**Figure 2:**
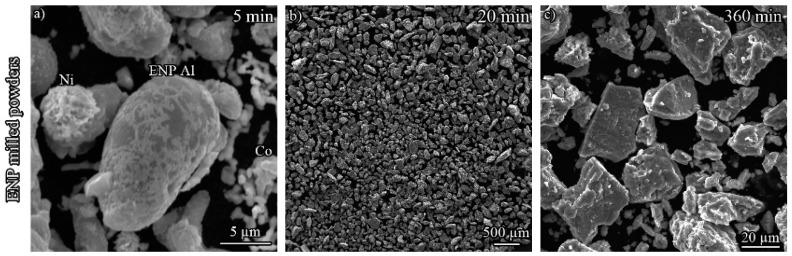

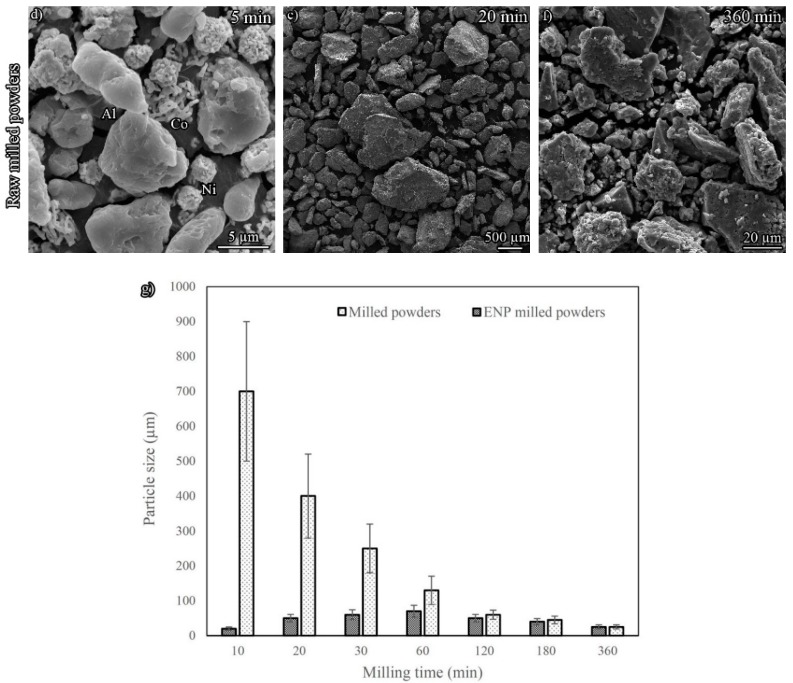
SEM images of particle evolution for both types of the specimens (**a**–**f**) and their mean size vs. time (**g**) during the milling process.

Figure 2 shows SEM images of the ENP (a–c) and raw (d–f) powders after various milling times. After 5 min of MA, a slight amount of ENP coating was removed from the particles (Figure 2a). However, the cold welding/fracture process of the powders was not initiated by this milling time (Figure 2a,d). With increasing the time to 20 min, colonies of the particles were formed. Nonetheless, there was a meaningful deviation in the size of the colonies for the raw and ENP coated powders. The mean size of the particles is 60 µm for the coated powder system, while it is 400 µm for another type of the specimen (Figure 2b,c).

The tendency of the particles to the repeated cold welding/fracture phenomenon is depicted by Figure 2g in terms of milling time and mean particle size during the process. It can be stated that this measure was higher for the coated powder mixture specifically at the beginning of the process. It was noticed that the deviation in mean particle size became smaller as the time increased. After 360 min of MA, there was no difference between the mean particle size of both types of the specimens (Figure 2c,f). The behavior of the raw powders is normal during the milling process once Al is the main elemental component of the mixture [9]. Consequently, this variation can be ascribed to the corporation of ENP coating as an anti-adhesive barrier comparing to bare Al particles in the raw powder mixture, preventing further growth of colonies at the beginning of the milling process.

**Figure 3:**
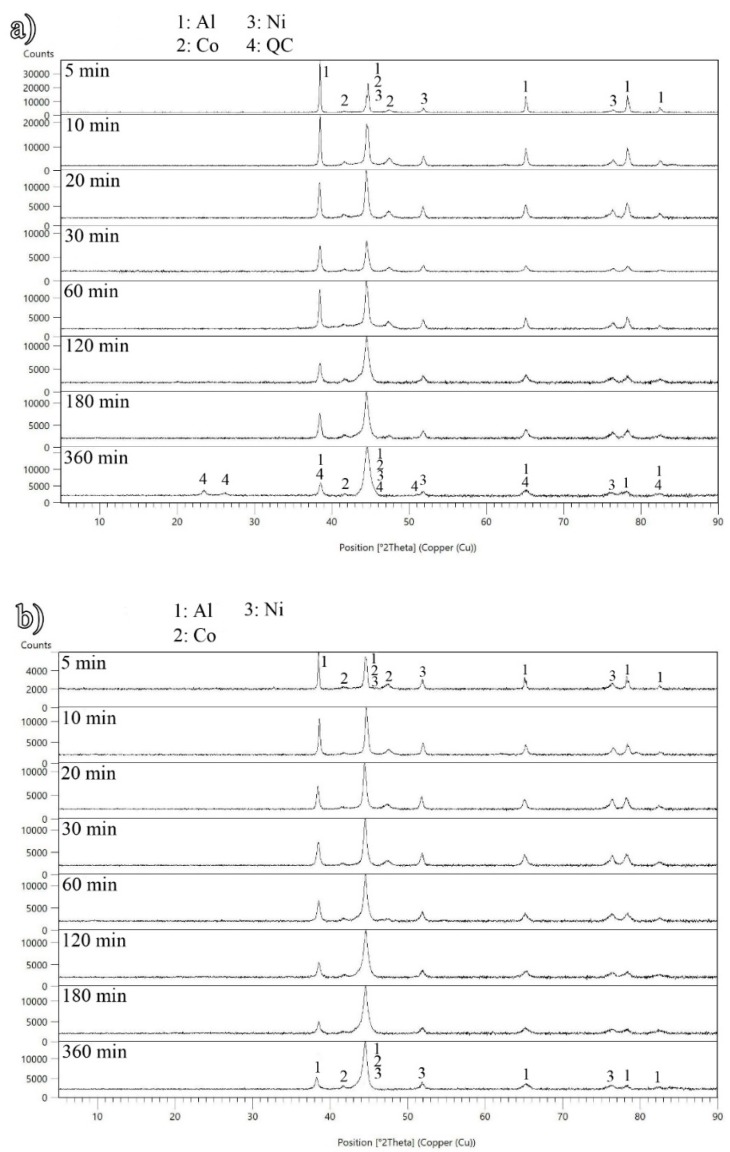
X-ray diffraction patterns of the Al_70_Ni_15_Co_15_ mixture after different milling times for the (**a**) coated and (**b**) raw powder specimens.

X-ray diffraction patterns of the coated and raw powder mixtures are shown in Figure 3a,b, respectively. The MA process on the raw powders resulted in no phase transformation. However, the elemental lines of the diffraction significantly broadened and reduced in intensity with increasing milling time. It happens due to a decrease in particle size and an increase in internal strains. These data indicate that the same evolution occurred in the coated powder mixture prior to 360 min of MA. In the final stage of the milling process, however, there was evidence exhibiting the formation of the QC phase in the vicinity of starting elements. Using the indexing scheme of Yadav et al. [3], the exclusive peaks of the QC phase in the coated powder mixture mechanically alloyed for 360 min can be identified as the (101100), (111100) and (111104) reflections as described in Table 1. Once the singular peaks of a new phase appear in the X-ray diffraction pattern, those which overlap with the peak positions of the initial elemental composition, may be also identified as the new phase. In this investigation, the other recognized QC peaks showed overlapping with starting elemental ones in 2θ = 38.47, 44.55, 65.27, and 82.11°. This is the reason why the indexation of the two types of specimens was different in 360 min of ball milling. Additionally, with the existence of QC peaks, the intensity of the elemental peaks diminished, specifically at the angles of 2θ = 41.88 and 47.45°. Using the well-known Williamson-Hall’s equation [10,11], the crystallite/quasicrystal size of the coated powders after 360 min of BM was calculated to be 17.7 nm, while the corresponding crystallite size was 55.5 nm for the raw powder system. This variation can be originated from the formation of a much harder QC phase in the coated powder system with increasing the milling time, which causes acceleration of reduction in crystallite/quasicrystal size.

**Figure 4:**
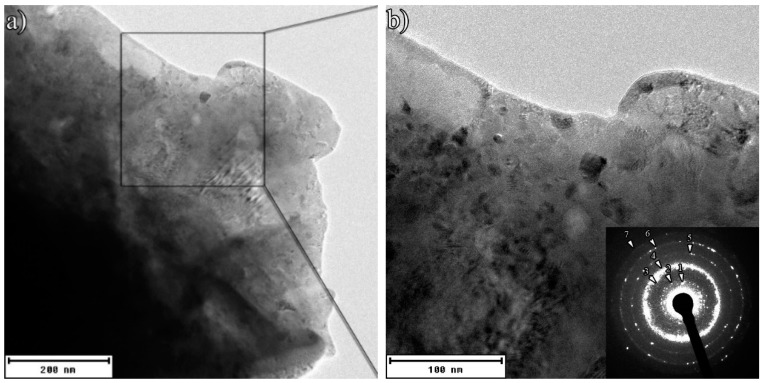
TEM images of the ENP coated powders after 360 min of MA at (**a**) lower and (**b**) higher magnifications.

Figure 4 illustrates the TEM images of 360 min ball milled sample (ENP coated) at lower and higher magnifications, exhibiting the formation of the nano-QC phase by the ring-type selected area diffraction (SAD) pattern [12]. The diffraction rings indexed to those of the decagonal phase. The corresponding indices (marked in the SAD pattern of Figure 4b) were previously described in Table 1. These features corroborate the interpretation of the X-ray results in terms of nano-QC structure formation.

**Figure 5:**
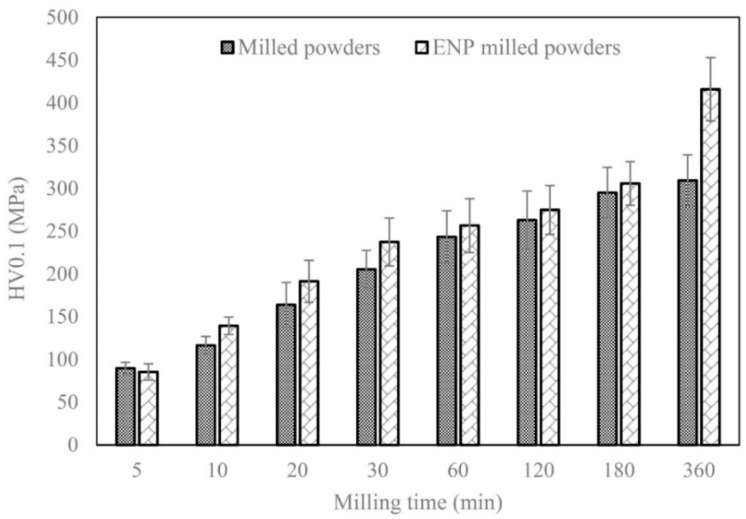
The variation in microhardness of both types of samples milled for different times.

Figure 5 exhibits the variation in the microhardness of the samples milled for various durations. With increasing milling time, the microhardness values of both types of samples were enhanced. However, these values were slightly higher for the coated powder samples in all given durations except 5 min of milling, which can be negligible as the deviation is very small. The reason may be attributed to the coated powders being subjected to a further repeated cold welding/fracture phenomenon during ball milling as discussed earlier (Figure 2g). It caused a further reduction in mean particle size and enhancement of the internal strains, leading to higher values of hardness. It is interesting to indicate that the difference between the hardness values of the samples milled for 360 min is meaningful. It can be attributed to the formation of nano-QC domains dispersed alongside the coated type specimens.

## 4. Discussion

Deformation induced transformations are very usual in many alloys during high-energy ball milling. For alloys with high stability, the required reaction temperatures for the formation of the QC phases may not be provided by the planetary ball mill. As is well known, a system is thermodynamically stable once the free enthalpy G is in its minimum amount. Generally, in the solid-state of metallic systems, the free enthalpy of the crystalline state is lower than those of the amorphous and QC phases [13]. In the present case, however, a remarkable amount of metallic Ni particles was replaced by the amorphous/solid solution Ni-P coating (Figure 1c). Therefore, the activation energy required for the transformation of amorphous to the QC phase is theoretically lower compared to direct transformation from the crystalline phase. In other words, the local temperature of the mixture may increase during the MA owing to the energy dissipation of the colliding balls, which is somehow sufficient to develop an interdiffusion reaction. For the experimental conditions used in this study, it was shown that the tendency of the coated particles for repeated cold welding/crashing is higher than that of raw powder mixture (Figure 2g). As a result, it may locally provide higher temperatures in the mixed powders and increase the possibility of an interdiffusion reaction, leading to the coexistence of QC and crystalline phases in the system.

One of the most prominent differences between the powder systems used in this study is the existence of phosphorus in the coated powder system caused by electroless Ni-P plating of initial powders. However, there is no report in the literature investigating the influence of phosphorus on the prohibition or promotion of QC formation. On the other hand, the role of this element in the formation of other phases is available [14,15]. As an example, a combined computational and experimental approach was applied by Ke et al. [15] to explain how minute amounts of phosphorus accelerate the ordering rate of stoichiometric Ni_2_Cr in Ni-Cr alloys. It was declared that the acceleration is caused by strong binding between phosphorus and vacancies in the applied alloy, which can increase the rate of phase transformation in a significant manner. In this investigation, the phosphorus may also have a positive effect on the formation of the nano-QC phases. Nevertheless, the mechanism of the action is still in question.

## 5. Conclusions

In this study, the formation of the QC phase in the Al-Ni-Co ternary system was assessed during the MA process. It was shown that the incorporation of ENP coating has a significant effect on the structural evolution of powders during the MA and consequently the formation of the QC phase in the vicinity of metallic powders. Moreover, the following conclusions can be drawn from the results.

- The incorporation of semi-amorphous Ni-P instead of metallic Ni in the AlNiCo powder system changed the behavior of the mixture during the primary steps of ball milling so that the mean size of particle colonies was several times smaller in the coated particle system compared to raw particle system.
- After 360 min of ball milling, the crystallite/quasicrystal size was 17.7 nm in the coated powder system, while it was 3.13 times higher for the raw powder system.
- Formation of the nano-quasicrystal phase in the coated powder system after 360 min of ball milling resulted in 34% enhancement of microhardness value compared to that in the raw powder system.

## Figures and Tables

**Table 1:** Indexing of electron diffraction pattern (QC phase) in Figure 3a.

Diffraction Ring No.	2θ	D Spacing (nm)	Indices
1	23.47	0.3787	101100
2	26.16	0.3403	111100
3	38.47	0.2338	102202
4	44.55	0.2032	211200
5	50.72	0.1767	111104
6	65.27	0.1428	211204
7	82.11	0.1173	303220
